# Getting everyone to the table: exploring everyday and everynight work to consider ‘latent social threats’ through interprofessional tabletop simulation

**DOI:** 10.1186/s41077-021-00191-z

**Published:** 2021-11-03

**Authors:** Ryan Brydges, Lori Nemoy, Stella Ng, Nazanin Khodadoust, Christine Léger, Kristen Sampson, Douglas M. Campbell

**Affiliations:** 1 Allan Waters Family Simulation Centre, St. Michael’s Hospital, Unity Health Toronto, 30 Bond St, Toronto, ON M5B 1W8 Canada; 2 Department of Medicine, University of Toronto, Toronto, Canada; 3 Technology Enabled Education, St. Michael’s Hospital, Unity Health Toronto, 30 Bond St, Toronto, ON M5B 1W8 Canada; 4 University of Toronto’s Centre for Interprofessional Education, University Health Network, Toronto, Canada; 5 Department of Speech-Language Pathology, University of Toronto, Toronto, Canada; 6 Department of Paediatrics, University of Toronto, Toronto, Canada

**Keywords:** Quality improvement, Healthcare systems, Interprofessional education, Institutional ethnography, Ethnography

## Abstract

In this methodological intersection article, we describe how we developed a new variation of the established tabletop simulation modality, inspired by institutional ethnography (IE)-informed principles. We aimed to design and conduct pilot implementations of this innovative tabletop simulation modality, which focused uniquely on everyday and everynight work, along with the factors that govern that work. In so doing, we aimed to develop a modality and preliminary findings that researchers and educators can use to simulate healthcare practices across longer episodes of care (i.e., time scales of hours or an entire day) and to detect the ‘latent social threats’ that can emerge during interprofessional clinical care.

An interprofessional team designed tabletop simulation scenarios of interprofessional challenges during transfers of care on a labour and delivery (L&D) unit. Within each scenario, participants provided real-time explanations for their work and associated drivers, both independently and as a team. Thus, we combined ‘think-aloud’ and simulation principles to design tabletop simulation scenarios to elicit healthcare professionals’ descriptions of how they collaborate in their work on the L&D unit. We completed a total of five tabletop simulations with eight participants (obstetricians, *N* = 2; midwives, *N* = 2; nurses, *N* = 5).

The conversations stimulated by the tabletop simulation scenarios and debriefs allowed us to generate a preliminary understanding of the texts that govern and organize clinicians’ everyday work processes. We generated data about longitudinal, multi-hour work processes in a condensed timeline, with opportunities to pause and probe, and with reduced focus on individual practitioner’s competence.

We believe our innovative tabletop simulation approach allowed us to examine clinical work in ways no other simulation permits. Participants described how the scenarios opened a productive dialogue between professional groups and suggested this simulation-based approach might contribute to enhanced interprofessional understanding and cultural change. We suggest that others can adapt our low-resource approach to understand clinicians’ everyday work and to map how this work is governed by documents, like policies, with the end goal of facilitating system change and managing latent social threats.

**Supplementary Information:**

The online version contains supplementary material available at 10.1186/s41077-021-00191-z.

## Background

Simulation programs often oscillate between identifying latent safety threats and delivering training that targets individual and team competencies [1–4]. In shifting between these two core activities, simulation educators and researchers may not consistently consider a key driver of individual and team work: the taken-for-granted everyday and everynight work of healthcare professionals’ and how it is organized by social or structural forces. For example, safety threats may arise from how professionals’ work is governed by the policies, guidelines, pay structures, laws, and regulatory pressures impacting their thinking about, as well as their actual scopes of practice.

Consequently, our team explored methodologies oriented toward the study of ‘work.’ We present this exploration as an innovation that followed a broader institutional ethnography (IE)-informed study [5]. That study focused on the work of midwives, obstetricians and nurses on an urban hospital’s labour and delivery (L&D) unit, which had formal links to a community midwifery centre. We borrowed concepts from IE scholars, who define ‘work’ as an activity that takes time, effort and intent [6]. IE researchers emphasize professionals as ‘informants’ about their everyday work, and focus specifically on what professionals do, rather than on their competency in or feelings toward work [7]. IE researchers typically aim their inquiry into how work is organized toward texts: the documents that function to connect everyday work with governing forces, such as policies, discourses, pay structures, laws, and/or models of care [8, 9]. IE’s purpose is to lead to social change.

Accordingly, after completing our IE-informed study, we sought to continue engaging the clinicians on the unit through structured co-learning activities (i.e., where researchers and participants / clinicians continue to learn together). With work as our construct of interest, learning more about how and what governs work as our purpose, and simulation as our area of expertise, we aimed to identify a simulation modality that would allow participants to detail their work processes. Tabletop simulation emerged as an ideal modality with potential to fulfill that purpose [2, 10–12]. Tabletop simulation is often used to understand systems-level disaster and emergency preparedness [13]. Generally, tabletop simulation scenarios involve participants working through a specific process using a physical blueprint of a space to identify deficiencies, inefficiencies, and barriers [13–17]. Thus, tabletop simulation often serves as a low-resource, bird’s eye “dry-run” of a large-scale process. We posited that the discussion-based format of tabletop simulation would align with our aim to probe professionals about their work, including the perceived organizational, legal, social, and cultural influences. This speculation, paired with previous successes when using tabletop simulations at our hospital [3], led us to design a novel tabletop simulation that aligned with our IE-informed aims.

In this methodological intersection article, we describe how we developed a new variation of the established tabletop simulation modality, as a response to our IE-informed study and insights from an interdisciplinary team. We aimed to design and conduct pilot implementations of this innovative tabletop simulation modality, which focused uniquely on everyday and everynight work, along with the factors that govern it. In so doing, we offer a modality and preliminary findings that researchers and educators can use to simulate healthcare practices across longer episodes of care (i.e., time scales of hours or an entire day).

## Methods

### Context

Previously identified challenges on our L&D unit during consults and transfers of care provided the need for which we designed our IE-informed research study, and the tabletop simulation approach presented below.

Inspired by our learning about IE principles in the preceding IE-informed study, we subsequently initiated this project to explore the feasibility and value of designing and implementing tabletop simulation scenarios focused on everyday and everynight work. Our data collection and analyses reported below did not, however, focus on gaining further insights about work and the texts that govern work on the L&D unit. Instead, we used our preceding experiences conducting the larger study as points of contrast for exploring the value-add, the ‘implementation fidelity’ (i.e., did we yield insights about work at all?), the lessons learned, and the challenges experienced in implementing an IE-informed tabletop simulation modality. In conducting both projects, we received and complied with research ethics board approval from our organization (Protocol # 17-358).

In the list below, we provide a brief summary of our IE-informed study findings to give further context regarding the hospital setting where we conducted our tabletop simulation scenarios: (i) we found that intersections of health and law provided the foundation for how the work of midwives, obstetricians and nurses was socially organized, (ii) that a medicine-centric governance of the unit and midwives’ practices appeared to perpetuate observed challenges, (iii) that the electronic fetal monitor (EFM) and how it was presented provided a ‘text’ that contributed to interprofessional conflicts, and (iv) that our efforts to make the multiple issues we uncovered on the unit explicit to the various healthcare professionals led to efforts toward collaborative, interprofessional change to unit policies and procedures [5].

### Piloting

We first piloted a tabletop simulation scenario (based on a previously observed case) with our research team that included intrapartum clinicians from the L&D unit: obstetricians (*N* = 2), nurses (*N* = 2), midwives (*N* = 2), a pediatrician (*N* = 1), and two family medicine physicians (*N* = 2). Feedback from the team prompted us to focus future scenarios less on medical ambiguity and thresholds for clinical management and more on the ambiguity of prolonged (over multiple hours) interprofessional interactions.

### Developing the scenarios

After receiving hospital REB approval, we designed three new scenarios. Each scenario probed a key interprofessional challenge during consults and transfers of care that emerged from the data we had previously collected through incident analysis team reports, field observations, and interviews within our IE-informed study [5].

### Simulation materials

**Fig. 1:**
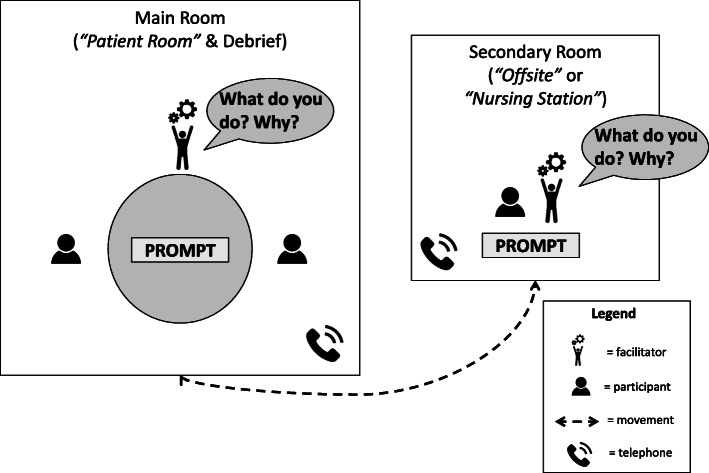
Example of tabletop simulation set-up and format

We needed two separate rooms to simulate staff working in different locations throughout the scenario (on-site, off-site, or elsewhere in the hospital) and two facilitators in each room (Fig. 1). Our simulation educators prepared clinical and content prompts on cue cards that they distributed throughout the simulation to progress each scenario.

### Sampling and recruitment

As the participants for our small data collection, we recruited clinicians involved in L&D unit decision-making, leadership, and educational administration. We sampled this population to probe varying perspectives and to collect feedback about how this modality might translate as a policy-informing or educational tool. We held simulation sessions with two different groups of clinicians and conducted two tabletop scenarios per session between May and September 2019.

### Conducting the simulation

**Fig. 2:**
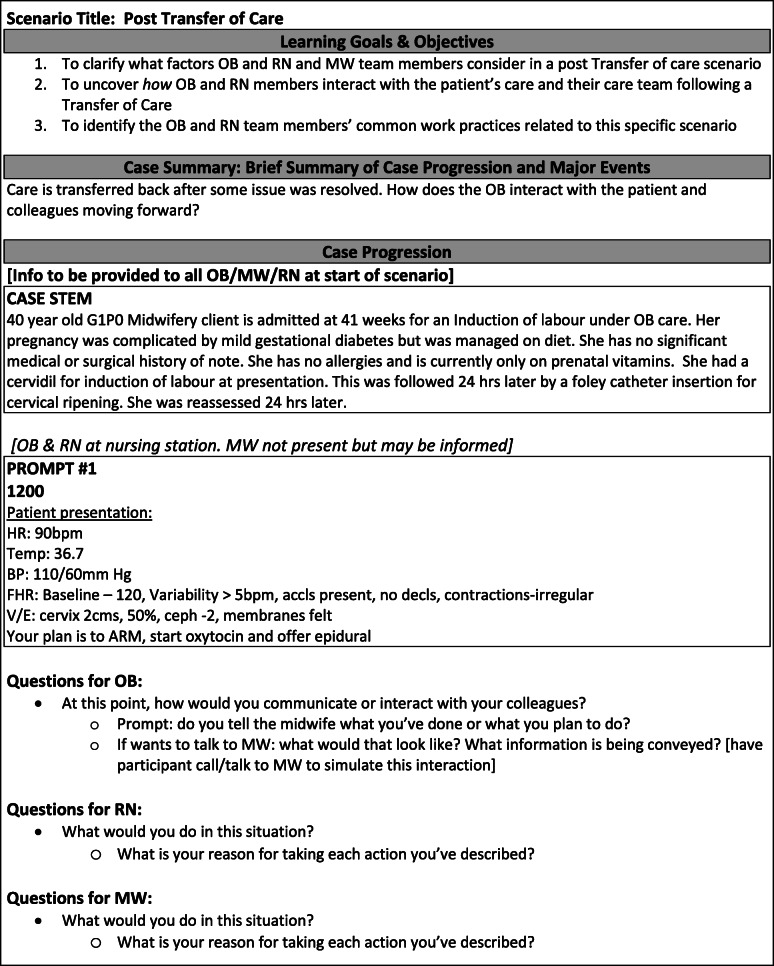
Sample tabletop simulation scenario excerpt

All participants were pre-briefed to expect being questioned about what they do and what prompts them to work that way in their everyday experiences (see Additional file 1). Following the pre-brief, all participants read a case stem (example in Fig. 2) and asked any immediate questions. Participants then dispersed to their respective locations (Fig. 1) to receive the first content prompts (example in Fig. 2). Each of these prompts accelerated the timeline of the scenario to moments where participants might typically decide to work interprofessionally (e.g., when a MW client’s OB-induced labour has progressed to the MW taking over care). Once participants explained what they would do in response to each prompt, the facilitators asked semi-structured follow-up questions to clarify what motivated them to take certain actions (i.e., 'why would you do it that way?'). Throughout the scenario, participants would move in and out of the rooms to simulate being in the same clinical space or apart from their colleagues (Fig. 1). As needed, participants would call each other by phone from the separate rooms to converse as usual. Simulation educators kept each other temporally-aligned by updating which time stamps their participants were currently working through via text-messages. Time-stamps on each prompt represented the progression of time in the scenario, enabling us to simulate a 24-h case in approximately 20 min.

After each scenario, the facilitators conducted an approximately twenty-minute semi-structured debrief, which borrowed from the “Promoting Excellence and Reflective Learning in Simulation” (PEARLS) framework [18].

### Field notes and reflective analysis

Throughout all the simulations, authors RB and LN were present to take field notes both during the scenarios and during the debriefs that followed. Once the participants left the simulation space, we met with the simulation educators (authors CN and KS) to discuss their insights and to document any adjustments or divergent points independently in our field notes. LN, RB, and DMC then met to synthesize their notes from across all the delivered scenarios. Next, LN, RB, and DMC presented their insights to the full research team at ongoing monthly meetings. Our collective reflections at these meetings were distilled into themes reflecting our perspectives on the value propositions of the tabletop simulation, the specific outputs generated for our simulation program team, and the lessons learned during design and implementation.

## Results

In addition to the pilot session, we completed a total of five tabletop simulations with eight participants (obstetricians, *N* = 2; midwives, *N* = 2; nurses, *N* = 5). Below, we focus on what we learned from the steps we took to design and implement our tabletop simulation scenarios.

### Innovation: “an interview-in-context”

In terms of ‘implementation fidelity,’ which we frame as whether the tabletop simulations generated data relevant to our IE-informed aims, we found that the simulations generated many insights about participants’ work. While not the central focus of our analysis in this article, participants talked in-depth about specific work practices during consults and transfers of care, which helped us form connections to texts that appeared to influence that work (e.g., scopes of practice defined by governing bodies, and policy documents within the hospital) [8]. In contrast to our experience conducting one-on-one interviews, we found that the tabletop scenarios more directly elicited rich data to inform our identification of those texts (i.e., participants noted relevant texts more explicitly). We also found that, when all three intrapartum participants were together in the tabletop scenario and debrief, they would respond to each other’s comments with counter-perspectives and rationales, which resulted in more context-rich responses than from the interviews.

As a specific example of one valuable insight, during a discussion about the central EFM of a patient under midwifery care, nurse participants spoke about how the EFM sounds an alarm at the nursing station if it detects concerning changes in fetal heart rate. These nurses expressed their strong feelings of discomfort regarding the medico-legal ambiguity of simultaneously not feeling they should involve themselves in that patient’s care, yet also feeling accountable given their name would be logged in the patient’s chart if they silenced the alarm. In this way, we learned how the EFM functioned as a text that influences how involved nurses feel and act in relatively rare intrapartum scenarios.

We attribute these data and merits to the situated nature of the tabletop discussion, which we came to view as an “interview-in-context.” That is, instead of asking participants to recall work that may feel routine and unremarkable to them, the actual scenario and direct questions about their work helped situate their perspectives and offered opportunities for reflection. Further, their colleagues’ presence served to prompt additional responses that an interviewer may not think to ask. These benefits facilitated our efforts to map some of the texts/documents that organize the work of consults and transfers of care.

### Generating rather than requiring a map

Educators often design tabletop scenarios using an existing map or blue-print, aimed at improving how work ought to be done. Our approach to tabletop simulation, conversely, did not use an existing map. Rather, it produced data that can be used to generate a map depicting a satellite view of everyday work, including how some actions divert from what is meant to happen, and which texts contribute to these diversions. As an example of what could be produced through systematic data collection, we refer interested readers to Fig. 1 in our IE-informed study [5]. Hence, our modified tabletop simulation may represent a tool for generating data to build maps that go beyond representing physical workflows toward uncovering ‘latent social threats’ to how work is done.

### New capacity and new resources

Immediate outputs included the three new tabletop scenarios that our simulation program and the L&D unit can use in future education and QI initiatives. The conceptualization, objective-setting process, and implementation of tabletop simulation were new to our simulation team and participating clinicians. As such, all required significant project coordination to design and implement. The consequence of this hard work, however, was that our team developed the capacity to design and implement such scenarios, and to innovate when adapting simulation modalities to novel challenges [4]. Further, this experience expanded our views of what constitutes ‘simulation,’ allowing us to be innovative, flexible and more intentional about how we select simulation modalities for education and research.

## Discussion

In this methodological intersection article, we aimed to capitalize on our team’s collective learning from conducting an IE-informed research study. After completing that study, we developed multiple tabletop simulation scenarios to address IE-informed objectives. We found that participants’ responses to our prompts during the scenario and subsequent debriefing taught us about their work and some of the texts governing it. Our adaptation of the tabletop simulation modality appears to have the potential to simulate longer episodes of care in the form of a rich interprofessional interview-in-context. In the sections below, we outline our lessons learned, recommend modifications to our approach, and consider the limitations of this innovation.

### Lessons learned, recommended uses, and modifications

We suggest three major modifications to our approach. First, where applicable, we would provide more visual prompts over narrative prompts (e.g., EFM tracings rather than summary numerical data of tracings), which would better reflect clinical practice and streamline participant responses to scenario prompts. Further, those developing tabletop scenarios might consider placing key texts explicitly into the scenarios, which may yield clear insights regarding how those texts contribute to participants’ work. As a note of caution, however, such explicit reference to documents may appear artificial to participants and/or could over-emphasize some texts to the detriment of learning more about the powers of others. Second, we would introduce a “facilitator” role to ask the probing questions and to lead the debrief, so simulation educators can focus on coordinating the logistics of the scenario (timing, distribution of prompts, movement between spaces). Third, for educators and researchers hoping to use tabletop simulations to generate data about everyday and everynight work and related texts, we recommend engaging with the key IE concepts that informed our study: work, standpoint, texts, and disjunctures [5]. Notably, even if these concepts are addressed well in any additional tabletop simulation scenarios, we caution that multiple IE-informed tabletop simulations would not amount to a robust IE study.

In that vein, we note that we borrowed specific ideas from IE inquiry in developing these simulations and did not engage deeply with IE as an approach. We focused on work and governing texts, which expanded our views of how simulation can generate data about and potentially impact health systems. Further work to incorporate an IE lens more comprehensively, as well as other methodologies from the social sciences will surely be beneficial. In its present form, we recommend that educators can adapt this low-resource tabletop approach to numerous contexts and functions; for example, as a stand-alone educational modality, as a form of program evaluation, as quality assurance or improvement, or as a complement to other simulation-based research data collection techniques.

Finally, we have played-on the concept of ‘latent safety threats’ to frame our thinking of an additional value-add of our IE-informed modality. Where ‘latent safety threats’ are commonly used to describe the unforeseeable physical malfunctions of equipment or spatial challenges that simulation can uncover [2, 3], we have begun to consider ‘latent social threats’ as the unforeseeable, or even unsee-able social factors that can contribute to error or communication breakdowns, like medico-legal concerns and the influence of policies, norms and culture. Further, we liken the term ‘latent social threat’ to the IE principle of disjunctures, which represent gaps between official representations of practice (e.g., a guideline) and people’s everyday experiences and uses of their knowledge [9]. Thus, during simulation scenarios and debriefs, any contradictions arising between participants in how work is ‘supposed to happen’ may represent a latent social threat worthy of attention (e.g., debates about when a transfer of care between professionals is appropriately triggered). While our conceptualization of latent safety threats will surely be refined, we recommend that the simulation community consider grappling with this additional type of safety threat beyond the current emphasis on physical workflows.

### Limitations

Given the accelerated timeline associated with condensing multi-hour episodes into twenty-minute scenarios, we missed the opportunity to capture uni-professional work occurring between the prompts. Further, we likely missed relevant data about the work associated with the travel and preparations required to care for a L&D patient safely and effectively. We also appreciate that recording participants’ descriptions of behaviour in a simulated context may not reflect their actual behaviour, which is why IE researchers emphasize a combination of direct observation and shadowing, immersion in the institutional context, interviews, textual analyses, and more [6, 8, 9]. Hence, as a scholarly approach, we recommend the tabletop simulation be viewed as a tool in the toolbox, rather than as a stand-alone method. Further, relative to other performance-based modalities, such as in situ and translational simulation, we note that the tabletop simulation modality involves extrapolating from what is said to form inferences about what would happen in actual practice. We suggest that this trade-off of accepting perspectives in place of observed behaviours was worth the gain of better representing the longer time scales associated with patient care, which the simulation community has previously struggled to develop in a cost-effective and resource-wise manner.

## Conclusions

Our use of IE concepts to inspire a novel tabletop simulation modality allowed us to capture and analyze clinicians’ descriptions of the what’s and why’s of their work without conflating it with their individual or team competencies. Indeed, we produced unique connections between clinicians’ perceptions of their everyday and everynight work and some of the texts governing and impacting that work. By uncovering these ‘latent social threats’, this tabletop simulation approach offers a way to collect data that identifies problematic social relations as targets for meaningful system change [6].

## Supplementary Information

**Additional file 1.** Briefing script for tabletop simulations

## Data Availability

The datasets used and/or analyzed during the current study are available from the corresponding author on reasonable request.

## Abbreviations

IE: Institutional ethnography
L&D: Labour and delivery
MW: Midwife
OB: Obstetrician
RN: Nurse
