# Supplementary material for: Getting everyone to the table: exploring everyday and everynight work to consider ‘latent social threats’ through interprofessional tabletop simulation
Source: Adv Simul (Lond). 2021 Nov 3;6:39. doi: 10.1186/s41077-021-00191-z (PMC8564977; doi:10.1186/s41077-021-00191-z)
Supplement: Supplementary file 1 — Additional file 1. Briefing script for tabletop simulations [file 41077_2021_191_MOESM1_ESM.docx]

**Briefing Script for Tabletop Simulations**

My name is XYZ and I am the research coordinator for the QUILT Study. To give you a brief introduction and to refresh your memory, the aim of this project is to understand the barriers and enablers to intrapartum teamwork and communication during transfer of care (ToC) and consults in the unit. We are trying to understand the factors that coordinate and influence your “everyday work” in relation to ToC and consults. One of the ways we are doing this is by running TABLETOP simulations where we will ask you to describe what action you would take as the scenario plays out. I will be asking probing questions to understand what might be shaping your work processes. If at times I sound like I’m looking for a specific answer, I’m not – this is not a *test* of your clinical judgment or skills, and I will not be judging your answers. I’m just trying to get you talking about your everyday work, and it will feel like I’m asking WHY a lot to do that. My questions are designed to make the work processes that are second-nature to you visible through our conversation so that we may learn from them to improve systems and structures. We will debrief the scenario immediately after its conclusion. The tabletop exercise will take roughly 15-20 mins, and the debrief will take 15-20 mins.

**Some reminders before we begin:**

1. We believe that everyone participating in simulation is intelligent, well trained, cares about doing their best and wants to improve. This is designed to be a **safe research environment.** Please be respectful at all times of everyone who is involved. We are all here to learn, and it is **okay** **to make mistakes**. We are not evaluating you in any way.
2. We have done our best to recreate a realistic scenario. We all acknowledge that this scenario is fictional, but please participate as you would in a **real clinical situation**. We recognize the challenge with tabletops is describing actions rather than doing them. Please try your best to describe your actions and processes thoroughly as you would normally do them.
3. “The Vegas Principle:” what happens in the simulation stays in the simulation. We keep our observations strictly confidential, and we ask that you do not share the content of this scenario or your colleagues’ actions or words (from the scenario or the debrief) with anyone else.
4. Please act in your current professional role throughout the activity.
